# The caspase-3/GSDME signal pathway as a switch between apoptosis and pyroptosis in cancer

**DOI:** 10.1038/s41420-020-00349-0

**Published:** 2020-10-28

**Authors:** Mingxia Jiang, Ling Qi, Lisha Li, Yanjing Li

**Affiliations:** Department of Gastrointestinal Oncology, Harbin Medical University Cancer Hospital, 150 Haping St, Nangang District, Harbin, Heilongjiang 150081 P. R. China

**Keywords:** Tumour-suppressor proteins, Oncogenes

## Abstract

Apoptosis has long been recognized as a mechanism that kills the cancer cells by cytotoxic drugs. In recent years, studies have proved that pyroptosis can also shrink tumors and inhibit cells proliferation. Both apoptosis and pyroptosis are caspase-dependent programmed cell death pathways. Cysteinyl aspartate specific proteinase-3 (Caspase-3) is a common key protein in the apoptosis and pyroptosis pathways, and when activated, the expression level of tumor suppressor gene Gasdermin E (GSDME) determines the mechanism of tumor cell death. When GSDME is highly expressed, the active caspase-3 cuts it and releases the N-terminal domain to punch holes in the cell membrane, resulting in cell swelling, rupture, and death. When the expression of GSDME is low, it will lead to the classical mechanism of tumor cell death, which is apoptosis. More interestingly, researchers have found that GSDME can also be located upstream of caspase-3, connecting extrinsic, and intrinsic apoptotic pathways. Then, promoting caspase-3 activation, and forming a self-amplifying feed-forward loop. GSDME-mediated pyroptosis is correlated with the side effects of chemotherapy and anti-tumor immunity. This article mainly reviews the caspase-3/GSDME signal pathway as a switch between apoptosis and pyroptosis in cancer, to provide new strategies and targets for cancer treatment.

## Facts

- As a key protein of apoptosis, caspase-3 can also cleave GSDME and induce pyroptosis.
- As a member of the Gasdermin family, GSDME plays an important role in the new pathway of pyroptosis, and its expression level determines the mode of tumor cell death.
- GSDME can be located not only downstream of caspase-3, but also upstream, connecting endogenous and exogenous apoptosis and promoting the activation of caspase-3.
- High levels of GSDME can increase the side effects of tumor chemotherapy.
- GSDME-mediated pyroptosis is related to anti-tumor immunity and tumor immune microenvironment.
- The occurrence of tumor pyroptosis provides a huge research prospect for tumor therapy.

## Open questions

- How to use tumor pyroptosis to increase the sensitivity of chemotherapy drugs and avoid their side effects?
- Does GSDME be an agonist of tumor cell death?
- How to promote tumor cells pyroptosis and avoid normal cells pyroptosis, thereby inhibiting the occurrence and development of tumor?
- What are the key signaling proteins that correlate closely with tumor pyroptosis and apoptosis?
- How to find a balance point to utilize caspase-3/GSDME-mediated pyroptosis for cancer treatment and anti-tumor immunity?

## Introduction

Cancer is a complex multifactorial disease with a high global mortality and a serious threat to human health. In addition, it is the principle death reasons in the developing countries and the second leading cause of death in the developed countries^1^. Its occurrence is mainly caused by abnormal cell growth which is induced by activation of proto-oncogene and inactivation of tumor suppressor gene. The imbalance between cell proliferation and cell death determines the rapid increase of tumor cells. In addition, tumor is also associated with chronic inflammation, oxidative stress, immune microenvironment, and other factors. At present, from the macroscopic aspect, cancer treatment mainly involves surgery, radiotherapy, and chemotherapy^2^. As we knew, cancer cell death mainly includes apoptosis, pyroptosis, autophagy, and necrosis. Among them, apoptosis plays an important role in the mechanisms. However, with the widespread of anti-cancer drugs, resistance to apoptosis has been defined as one of the hallmarks of cancer and has been demonstrated to play an irreplaceable role in chemoresistance. Thus, no clear and effective treatment for tumors has been found. Tumors are still extremely aggressive and patients have a low survival rate^3^.

Therefore, scholars began to conduct in-depth research on cell death modes other than apoptosis, and the newly discovered pyroptosis gradually came into the field of scholars. Pyroptosis and the relationship between pyroptosis and apoptosis were further studied, and a common key protein, cysteinyl aspartate specific proteinase-3 (caspase-3), was identified, as well as Gasdermin E (GSDME), which determines the mode of cell death. In this mini-review article, we mainly introduce the changes of tumor cell death mechanism mediated by caspase-3 in different GSDME expression levels. GSDME can be used as a switch molecule in the transformation of apoptosis and pyroptosis. When it is highly expressed, cytotoxic drugs can induce tumor cell death through caspase-3-dependent pyroptosis. When the expression is low, the cell death mode is changed to apoptosis^4,5^. By taking this as an entry point, scholars hope to explore the mechanism of tumor development and treatment, and to propose new targets and strategies for clinical treatment. Interestingly, recent studies have found that GSDME has a pro-apoptotic effect in addition to its membrane pore effect. However, GSDME is mainly highly expressed in normal cells, and is low in tumor cells due to the hypermethylation of its promoter. When the expression of GSDME was low in tumor cells, the DNA methyltransferase inhibitor Decitabine could inhibit the hypermethylation of its promoter, thus further promoting the pyroptosis of tumor cells^4–6^. This review will summarize and discuss the potential effects of caspase-3 dependent cell death on cancer and the side effects of chemotherapy in caspase-3-dependent cell death with high expression of GSDME.

## Caspase-3

The caspase family is a protein family highly homologous to *C. elegans* cell death abnormal-3 gene (CED-3)^7^. Caspases can be divided into initiator caspases (caspase-2, -8, -9, -10), executioner caspases (caspase-3, -6, -7) and inflammatory caspases (caspase-1, -4, -5, -11)^8^. Loss of caspase activity is an important cause of tumor progression. As a member of the caspase family, caspase-3 was cloned from human Jurkat T lymphocytes by Fernandez-Alnemri et al. in 1994. It was originally named cysteine protease protein, 32kD (CPP32) because it encodes a 32kD cysteinyl aspartate specific proteinase^9^. Caspase-3 exists as an inactive proenzyme in the cytosol and performs functions by catalyzing the C-terminal cysteine residue to specifically lyse the peptide bond following aspartic acid residues. Caspase-3 was cleaved by granzyme B or caspase-10 at the D175 site. Then, p20 and p11 subunits were composed which induced the activation of caspase-3. Caspase-3 could not be activated by self-splicing or autocatalysis. Activated caspase-3 can degrade intracellular structural proteins and functional proteins and induce cell death^10^.

Compared with other members of the caspases family, caspase-3 is at the end of the caspase cascade and activated by both the intrinsic and extrinsic death pathways in apoptosis. And in recent years, the another effects of caspase-3 have been found, it can be located upstream of GSDME and play an inflammatory cutting role in pyroptosis^5^. It can be concluded that caspase-3 has multiple effects on the mechanism of tumor cell death compared with others. Accordingly, the following describes the research on caspase-3 dependent cell death in recent years. Many scholars have used caspase-3 as the starting point, with a view to understanding the mechanism of tumor cell death, apoptosis resistance, and pyroptotic tumor suppressor.

## Caspase-3-dependent cell death pattern

### Apoptosis

Apoptosis is a non-inflammatory form of programmed cell death (PCD) mediated by activation of the apoptotic caspases and can occur either via an intrinsic or an extrinsic pathway^4^. The intrinsic pathway is activated by mitochondrial damage. Subsequently the cytochrome c release into the cytoplasm from the mitochondrion, combines with apoptotic protease activating factor-1 (Apaf-1) and a caspase-9 precursor form an apoptosome that activates caspase-9^11^. Activated caspase-9 then cleaves and activates pro-caspase-3/7, leading to cell death by cleaving different cellular endogenous substrates. The extrinsic pathway is activated by cell surface death receptors signals, such as tumor necrosis factor-α (TNF-α) binds to death receptors, then the oligomerization of these receptors lead to the recruitment and activation of caspase-8, which directly cleaves pro-caspase-3 to mediate apoptosis^12–14^. Furthermore, caspase-8 can also cleave Bid, a member of the Bcl-2 (B-cell lymphoma 2) family, producing a truncated segment, tBid, which migrates to the mitochondria and forms Bax/Bak pores on its surface, releasing cytochrome c and activating apoptosis. In summary, activated caspase-3 is located at the end of the caspase cascades, activated by endogenous and exogenous apoptotic pathways, and is considered to be a key protein for apoptosis^15,16^.

The activation of caspase-3 leads to plasma membrane blabbing, chromatin condensation, DNA cleavage, and exposure of phosphatidylserine on the extracellular side of the plasma membrane^17^. Thus, produced the morphological and biochemical characteristics of apoptotic cells. In cancer treatment, many chemotherapeutic agents are thought to exert their cytotoxic effects on tumor cells through the induction of apoptosis. However, the lack of caspase activity caused by inhibitors and the changes and mutations of caspases in cell signaling pathways may inhibit the occurrence of apoptosis^18,19^. Furthermore, the imbalance between cell division and death will make mammalian tumors lose their promoters and the ability to execute apoptotic death procedures, which ultimately cause tumors to be resistant to the cytotoxic drugs^20^. Recently studies have confirmed that pyroptosis, as an emerging inflammatory cell death mode, provides the possibility to further reduce the side effects and drug resistance of chemotherapy^21,22^. Accordingly, academicians began to conduct in-depth research on pyroptosis and the relationship between pyroptosis and apoptosis to improve the current situation of cancer treatment.

### Pyroptosis

**Fig. 1:**
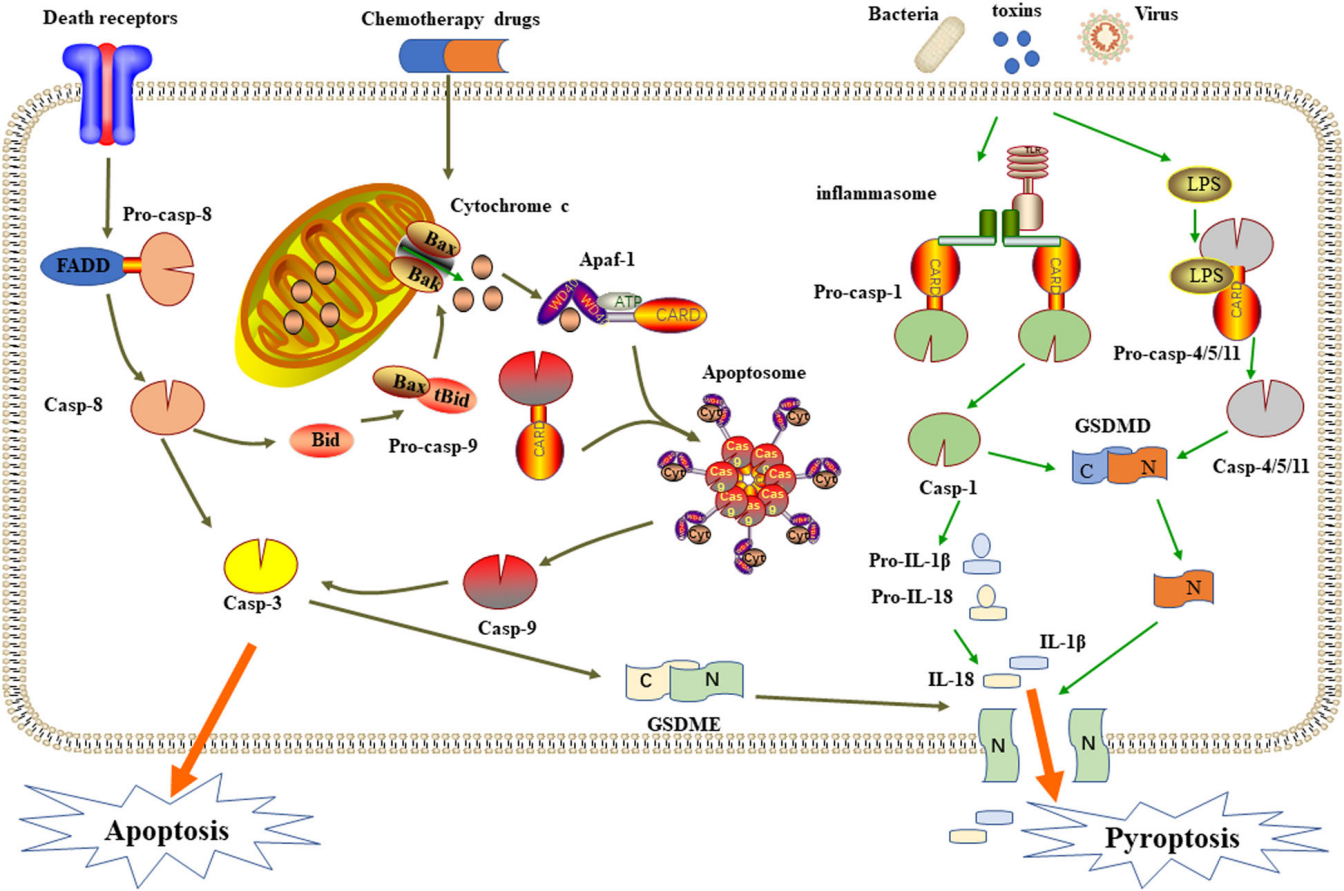
Molecular mechanism to caspase-3 dependent cell death and schematic diagram of the classical and non-classical pathways of pyroptosis. The route includes apoptosis consisting of endogenous and exogenous pathways and the newly discovered caspase-3/GSDME-mediated pyroptosis pathway.

Pyroptosis is a form of pro-inflammatory cell death that relies on the caspase family, and is one of the PCD mode. The earliest observation of pyroptosis can be traced back to 1986, when Arthur Friedlander et al. treated primary mouse macrophages with anthrax lethal toxin, resulting in cell contents to be released quickly^23^. In 2001, Cookson et al. first-named caspase-1-dependent PCD as pyroptosis^24^. At the outset, pyroptosis can be divided into caspase-1-dependent classical pathway and caspase-4/5/11 dependent non-classical pathway. The effector protein of caspase-1/4/5/11 is Gasdermin D (GSDMD). The activated caspase cleaves the 53 kDa GSDMD to form an N-terminal domain with 31 kDa, thereby forming pyroptosis. In addition, in the non-classical pathway, caspase-4/5/11 needs to directly recognize the lipopolysaccharide in the cytoplasm through the caspase activation and recruitment domain (CARD) domain^25^. In GSDMD-dependent pyroptotic pathway, there are mainly caused by stimulation of various abnormal signals, including crystalline substances, cytotoxins, adenosine triphosphate (ATP), and so on. In recent years, the caspase-3/GSDME-dependent pyroptosis signaling pathway has been found. GSDME located on the down-stream of activated caspase-3 forms N-terminal domains that recognize and punch holes in the cell membrane, causing cell swelling and rupture, releasing inflammatory factors and damage-associated molecular patterns (DAMPs) (Fig. 1)^25–27^.

Not only can pyroptosis cause abnormal cell death, but also recruit immune cells to trigger an inflammatory cascade that leads to inflammatory death of normal cells. Pyroptosis has been found to be a secondary necrosis after apoptosis, and was the result of the progressive loss of plasma membrane integrity of apoptotic cells. Therefore, pyroptosis has attracted people’s attention in the field of tumor. More and more researches have explored its role in tumor cell death. This mini-review mainly introduces the anticancer effect of pyroptosis in cancer treatment.

## The effector protein of caspase-3 in pyroptosis: GSDME

The Gasdermin family is an important protein that mediates pyroptosis, it can induce cell death and promote the release of inflammatory factors. It is mainly composed of six members: Gasdermin A (GSDMA), Gasdermin B (GSDMB), Gasdermin C (GSDMC), GSDMD, GSDME, and DFNB58^28^. Family members have 45% sequence homology, and most members have highly similar protein domains^29^. When pore-forming domains are activated, pores can be formed in the cell membrane to induce pyroptosis. Among them, besides GSDMD, GSDME has been the most widely used in the study of pyroptosis of tumor cells in recent years.

GSDME is expressed in cochlear hair cells and is found to be the result of the development of senseogenic deafness due to mutations in the gene intron 7 that lead to the jump of exon 8, which introduces an premature stop codon, resulting in the premature termination of the open reading frame and the translation of the C-terminal truncated protein^14,30^. GSDME is highly expressed in normal tissues, and is known to be a candidate tumor suppressor. It is low expressed in tumor tissues due to the hypermethylation of promoter and is only highly expressed in certain cancer tissues such as renal carcinoma, lung cancer, breast cancer, skin melanoma, and esophagus cancer^31^. It is a transcriptional target of p53 and is silenced in different cancers^32,33^. GSDME can be used as a key protein in the conversion of apoptosis and pyroptosis. In cancer cell lines with high GSDME expression, such as SH-SY5Y cells and MeWo cells, scholars identified a caspase-3-cleavable site _267_DMPD_270_ in human GSDME^5^. Then, the chemotherapy drug, as an activator of caspase-3, can induce activated caspase-3 which cleaves GSDME instead of PARP. GSDME-N produced by this process, can act on the plasma membrane of apoptotic cells to form pyroptotic-like necrosis, and deliver immunogenic effectors^4,34^. Hu et al. found that drug-induced cancer cells pyroptosis through the GSDME-C palmitoylation and the membrane hole effect of GSDME-N^35^. Loss of GSDME has been shown to abrogate the effectiveness of some chemotherapeutic drugs. From this, studies confirmed that in cancer tissues with low GSDME expression, the DNA methyltransferase inhibitor Decitabine can inhibit the hypermethylation of the GSDME promoter and increase the expression of GSDME, then promoting the occurrence of pyroptosis^4–6,36^. In summary, it can be concluded that GSDME has an important effect on the mechanism of tumor cells death. The following reviews the role of caspase-3-mediated cell death in cancer treatment at different GSDME expression levels in recent years.

## Assosiation between caspase-3 dependent cell death and cancer

### Lung cancer

When GSDME is highly expressed, both Cisplatin and Paclitaxel can activate caspase-3 to cut GSDME, and transform the cell death pathway from apoptosis which induced by traditional chemotherapy drugs to pyroptosis, and inhibit the proliferation of lung cancer cells^37^. In addition, the treatment of lung cancer with the Piperlongumine analogue L50377 or incorporation of an α,β-unsaturated ketone unit into chalcone can increase reactive oxygen species (ROS), promote the activation of caspase-3, incise GSDME, and induce pyroptosis^38,39^. Other recent studies have shown that after 28 h of the treatment of a new thiopyran derivative, L61H10, GSDME was cleaved by caspase-3. In addition, L61H10 can inhibit the degradation of IκBα (an inhibitor of NF-κB) induced by TNFα in H460 cells. After transfection with the IKKβ (an important kinase of the NF-κB signaling pathway) plasmid, the NF-κB pathway was activated. Then the anti-tumor effects of L61H10 were downregulated, apoptosis and pyroptosis were inhibited. Therefore, Chen et al. preliminarily confirmed that inhibition of NF-κB by L61H10 may cause an apoptosis-to-pyroptosis switch, but further studies are needed^40^. According to the latest research, Lu et al. extended the traditional view that apoptosis is the only death pathway for molecular targeted therapies, and established a potential clinical correlation between GSDME expression and the pyroptotic process of lung cancer. They demonstrated that by activating the mitochondrial intrinsic apoptotic pathway, molecular targeted therapies facilitated caspase-3 cleavage of GSDME to elicit pyroptotic cell death. From a therapeutic perspective, pyroptosis in cancer can enhance the sensitivity of molecular targeted therapies and overcome drug resistance^41^.

### Melanoma

A combination of BRAF and MEK inhibitors is commonly used in the treatment of melanoma. Researches have shown that BRAFi + MEKi treatment induces caspase-3 activation and increases cleavage of caspase-3, which promotes the production of the 35 kDa GSDME cleavage fragment in mouse and human melanoma cells^42^. Yu et al.^43^ showed that the inhibition of calmodulin-dependent protein kinase III (eukaryotic elongation factor-2 kinase,eEF-2K) can enhance the pyroptosis-promoting effect of Doxorubicin on melanoma cell lines with high GSDME expression. However, there was no pyroptosis in breast cancer cell lines with low expression of GSDME. Another study confirmed that abundant amounts of iron in combination with clinical drugs can activate the production of ROS, causing the oxidation and oligomerization of the mitochondrial outer membrane protein Tom20. Bax is recruited to mitochondria by oxidized Tom20, which facilitates cytochrome c release to cytosol to activate caspase-3, eventually triggering pyroptotic death by inducing GSDME cleavage^44^. However, when GSDME expression was low, Liu et al.^45^ found that Shikonin, which can act on melanoma cells and activate caspase-3 in a time-dependent manner to induce apoptosis. Increased expression of HMGB1 is known to be correlated with the progression of human cutaneous melanoma and poor patient survival. Studies have confirmed that a member of the Saururaceae family, Houttuynia cordata Thunb (HCT), can induce the activation of caspase-8 and caspase-3, activate p38, and MAPK proteins to induce apoptosis, and inhibit the release of HMGB1^46^. Above all, pyroptosis has an obvious therapeutic effect in melanoma cells. It can inhibit the proliferation of melanoma cells. Meanwhile, pyroptosis can also release inflammatory factors such as HMGB1 that are highly expressed in human melanoma, and promote the progress of melanoma. Therefore, the role of pyroptosis in cancer still needs further study.

### Osteosarcoma

Ding et al. demonstrated that a steroidal saponin derived from medicinal plants, Dioscin, can induce apoptosis and pyroptosis of osteosarcoma cells. By reducing mitochondrial potential, the expression of cytochrome c and pro-apoptotic protein Bax were upregulated, and the anti-apoptotic protein bcl-2 was downregulated. Dioscin can also enhance the expression of GSDME-N which is generated by caspase-3, and induce pyroptosis. In addition, knockdown of GSDME reduced the proapoptotic effect of Dioscin on OS^47^.In another study, Rogers et al.^14^ also found that the role of GSDME in targeting the mitochondria to promote caspase-3 dependent apoptosis, and the GSDME-N terminal domain can be located on cardiolipin on the mitochondrial membrane, forming a membrane pore effect, leading to the release of cytochrome-c, and then amplifying the internal signaling pathway of apoptosis. Wen et al.^48^ found that a lanostane type triterpenoid isolated from Poria cocos, Pachymic acid (PA),can suppress cell proliferation by the induction of caspase 3-mediated apoptosis in an immortalized cell line (HOS) and primary osteosarcoma. Li et al.^49^ claimed that Bcl-2 might be a direct target of miR-143. High expression of miR-143 can inhibit the expression of Bcl-2, and cause the activation of caspase-3, then induce apoptosis with upregulation of PARP, Bax, Bak, and Bad.

Collectively, caspase-3 is closely related to osteosarcoma cells. Moreover, the expression of GSDME determines the death mechanism of tumor cells. It can not only cause pyroptosis, but also show pro-apoptotic effects. In-depth study of the relationships about osteosarcoma and caspase-3 and GSDME are helpful for cancer treatment.

### Digestive cancers

Colon cancer is one of the most common malignancies of digestive cancers. Studies on colon cancer have found that when used Lobaplatin can activate caspase-3 to enhance pyroptosis, and in parallel to activate the ROS/JNK/Bax mitochondrial apoptosis pathway which increases the release of cytochrome c. The release of cytochrome c formed a positive feedback amplification loop to increase the activation of caspase-3^50^. The polypyrimidine tract-binding protein 1 (PTBP1), also named heterogeneous nuclear ribonucleoprotein I, is a widely studied splicing regulatory protein. Li et al. studied the biological function of PTBP1 in colon cancer cells and discovered that when PTBP1 is downregulated, Bax, cytochrome c, and p53 can be upregulated, and then apoptosis-related proteins caspase-3 and PARP1 are activated, thereby inducing apoptosis^51^. Wu et al.^52^ unveiled that Bufalin which is extracted from the skin glands of Bufo gargarizans or Bufo melanostictus, could significantly induce apoptosis in HCT-116 and SW620 colon cancer cells via mitochondrial ROS-mediated caspase-3 activation. Recently, Hu et al. found that TNFα + CHX and navitoclax-induced colon cancer cells pyroptosis through a BAK/BAX-caspase-3-GSDME signaling pathway, which promotes GSDME to be palmitoylated on its C-terminal (GSDME-C). Meanwhile, 2-bromopalmitate (2-BP) could inhibit the GSDME-C palmitoylation and chemotherapy-induced pyroptosis^35^.

Esophageal cancer is the eighth most common malignancy in the world^53^. Due to progressive dysphagia, esophageal stenosis, and high resistance to radiotherapy and chemotherapy, it often lost good efficacy^54^. Studies have found that the use of BI2536 which is an ATP-binding domain inhibitor of Polo-like kinase-1 (PLK1), in combination with Cisplatin on esophageal cancer cells can enhance the activity of caspase-3, cause pyroptosis, and in parallel to enhance DNA damage^55^. In addition, Tang et al. found that Metformin, a drug widely used in type 2 diabetes, could reduce the expression of Pyruvate kinase isozyme type M2 (PKM2) in esophageal cancer cells. And the downregulation of PKM2 can activate caspase-3, increase the expression of pro-apoptotic protein Bim. Eventually, apoptosis of esophageal cancer cells was induced and the prognosis of cancer was improved^56^. Liu et al.^57^ found that Epigallocatechin-3-gallate (EGCG), which was a major catechin in green tea with anticancer, antioxidant, and immunomodulatory effects. It can induce apoptosis of esophageal cancer cells, decrease the bcl-2 protein expression and increase the expression of Bax and caspase-3 protein.

Gastric cancer is a malignant tumor with the fifth incidence and third mortality worldwide^58^. Because most patients are often diagnosed at an advanced stage, when surgery is not recommended, resulting in a low five-year survival rate of people^59^. High expression of GSDME in gastric carcinoma cells can convert caspase-3 which induces both apoptosis and pyroptosis after treated with chemotherapy drugs such as 5-FU in GC cells. Scholars found the swelling cells, large bubbles from the plasma membrane, released LDH, decreased cell viability, and elevated percentage of PI and APC Annexin-V double-positive cells^60–62^. Li et al.^63^ noted that the flavonoids extracted from the Silybum marianum in the composite family, Silibinin, can induce endogenous apoptotic pathways through the mitochondrial pathway, and activate the ultimate executor of apoptosis,caspase-3, thereby inhibiting the proliferation and migration of gastric cancer cells.

### Other cancers

Osthole is a traditional Chinese herbal medicine used to treat for gynecological disease, nephritis, and ringworm. Recently, study from Liang et al. have showed that Osthole could induce ovarian cancer (OC) cells death through a variety of mechanisms. It can induce the OC cells apoptosis through reducing mitochondrial membrane potential, producing ROS, and eventually activating caspase-3. Osthole can also induce LC3-mediated autophagy in the OC cells. Furthermore, when GSDME is highly expressed in the OC cells, it can induce cell pyroptosis mediated by caspase-3^64^.

Glioblastoma multiforme (GBM) is the most common primary CNS tumor with a very aggressive course and poor prognosis^65,66^. Galangin (GG), a flavonoid, elicits a potent antitumor activity in diverse carcinomas. Kong et al. have examined the role of caspase-3 dependent apoptosis and pyroptosis, two mechanisms that induce PCD, in GG-induced inhibition of human GBM cell growth. GG simultaneously induces apoptosis, pytoptosis, and protective autophagy in GBM. By inhibiting protective autophagy, scholars have found that they can enhance the apoptosis and pyroptosis of GBM cells induced by GG. Compared with GG alone, when treated GBM cells with GG combined with the autophagy inhibitor, Chloroquine, can inhibit tumor growth, and improve survival rate. In addition, research has revealed that the two caspase-3 dependent cell death mechanisms may interact for efficient execution of cell death in response to treatment. Therefore, further research on the two death mechanisms and key proteins on the pathway is needed in the future, in order to improve the current treatment of cancer^67^.

Collectively, the drugs can induce tumor cells to undergo caspase-3 dependent cell death (Table 1). The expression of GSDME is closely related to caspase-3 dependent cell death. When it is highly expressed, pyroptosis occurs. Because chemotherapy resistance is closely related to the anti-apoptosis of tumor cells, it is urgent to further study the mechanism of tumor cell death and improve the treatment and prognosis of cancer. Therefore, scholars can conduct in-depth research from the perspective of caspase-3 to provide new targets and strategies for cancer treatment.

**Table 1:** Caspase-3-dependent cell death patterns in tumors.

Drugs	Cancer types	Mechanism	Ref
Cisplatin and Paclitaxel	Lung cancer	Activate caspase-3/GSDME	^37^
Piperlongumine analogue L50377	Lung cancer	Promote ROS and activate	^38^
Chalcone analogue	Lung cancer	Promote ROS and activate caspase-3/GSDME-mediated pyroptosis	^39^
Thiopyran derivative L61H10	Lung cancer	Cause an apoptosis-to-pyroptosis switch via NF-κB/GSDME	^40^
BRAFi + MEKi	Melanoma	Activate caspase-3/GSDME	^42^
Doxorubicin	Melanoma	Activate caspase-3/GSDME-mediated puroptosis via eEF-2K	^43^
iron	Melanoma	Activate Tom 20/Bax/cytochrome c/caspase-3/GSDME	^44^
Shikonin	Melanoma	Induce apoptosis by activating caspase-3	^45^
Houttuynia cordata Thunb	Melanoma	Cause the activation of caspase-8/3 and p38/MAPK	^46^
Dioscin	Osteosarcoma	Activate caspase-3/GSDME and upregulate cytochrome c/Bax	^47^
miR-143	Osteosarcoma	Target the Bcl-2 directly and activate caspase-3	^49^
Pachymic acid	Osteosarcoma	Induce apoptosis by activating caspase-3	^48^
Loboplatin	Colon cancer	Promote ROS/JNK/Bax/cytochrome c mitochondrial apoptosis pathway and activate caspase-3/GSDME	^50^
PTBP1	Colon cancer	Upregulate Bax/cytochrome c/p53 and caspase-3/PARP1	^51^
Bufalin	Colon cancer	Promote ROS and activate caspase-3	^52^
TNFα + CHX and navitoclax	Colon cancer	Through a BAK/BAX-caspase-3-GSDME signaling pathway to promote GSDME-C terminal to be palmitoylated, and induce pyroptosis	^35^
5-Fu	Gastric cancer	Activate caspase-3/GSDME	^60–62^
Silibinin	Gastric cancer	Activate caspase-3 and induce endogenous apoptotic pathways through the mitochondria	^63^
PLK1 inhibitor BI2536	Esophageal cancer	Combine with Cisplatin, activated Bax/caspase-3 and caused an apoptosis-to-pyroptosis switch via GSDME	^55^
Metformin	Esophageal cancer	Downregulate PKM2 and induce apoptosis by activating caspase-3/Bim	^56^
EGCG	Esophageal cancer	Induce apoptosis by downregulating Bcl-2 and upregulating caspase-3/Bax	^57^
Osthole	Ovarian cancer	Promote ROS and activate caspase-3 which induce apoptosis and pyroptosis	^64^
Galangin	Glioblastoma multiforme	Activate caspase-3/GSDME Combine with chloroquine, improved the effectiveness of cancer therapies	^67^

## GSDME-mediated pyroptosis and tumor immunity

Immunotherapy has revolutionized the treatment of cancer, and is a treatment method that restores the body’s normal anti-tumor-immune response by restarting and maintaining the tumor-immune cycle, thereby controlling and eliminating tumors^68^. It is not only to activate the immune system against tumors, but also to take account of immunosuppressive tumor microenvironment^69^. Among them, cytotoxic lymphocytes such as natural killer (NK) cells and cytotoxic T lymphocytes (CTLs or CD8^+^ T cells), play an important role in immunotherapy.

**Fig. 2:**
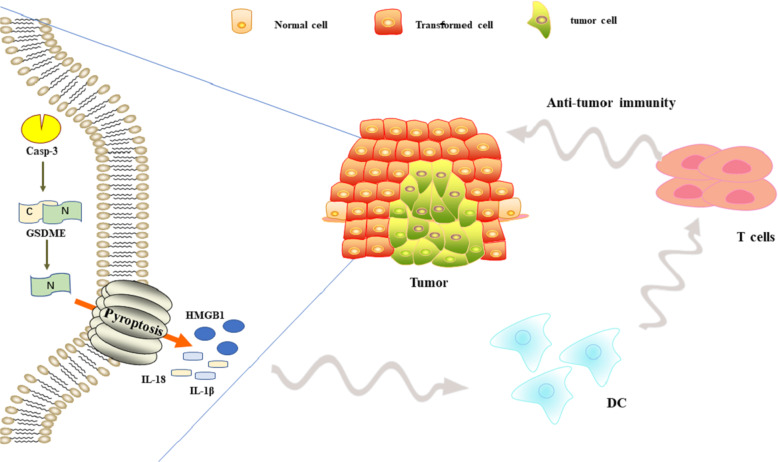
Correlation between pyroptosis and anti-tumor immunity. Pyroptosis can enhance anti-tumor immunity and exert anti-tumor effects.

Target cell killing by cytotoxic lymphocytes is primarily mediated through the release of cytotoxic granules that contain granzymes and perforin. Recent studies have found that both killer-cell granzyme B and caspase-3 use cleavage at D270 to activate GSDME, induce tumor cells pyroptosis, enhance anti-tumor immunity, and exert tumor suppression effects. The expression of GSDME enhances the phagocytic function of tumor-associated macrophages on tumor cells, and enhances the number and functions of tumor-infiltrating NK cells and CD8^+^ T cells^70,71^. Similar studies have also found that granzyme A in cytotoxic lymphocytes can cleave the GSDMB of tumor cells to induce pyroptosis^72,73^. Targeted drugs induce GSDME-mediated pyroptosis in melanoma, linking the tumor-immune microenvironment with T cells-mediated anti-tumor immunity^42^. In summary, GSDME-mediated pyroptosis plays an important role in cancer treatment and anti-tumor immunity (Fig. 2). In-depth study of it will provide new perspectives for cancer treatment.

## High-level GSDME increased the side effect of chemotherapy

As can be seen from the above, GSDME-mediated pyroptosis can kill tumor cells. Some scholars have suggested that during the treatment of malignant tumors, appropriate chemotherapeutic drugs can be selected according to the expression of GSDME, thereby increasing the sensitivity to chemotherapeutic drugs, enhancing the anti-tumor immunity and reducing drug resistance^74^. However, some scholars also proposed that the high expression of GSDME would enhance the side effects of chemotherapy.

As is well known, most of GSDME is expressed in various normal tissues, and is absent in most tumor cells. So, does pyroptosis induced by chemotherapy drugs kill normal cells? Does the expression of GSDME increase the adverse effect of chemotherapy drugs? Wang et al.^5^ found that several primary human cells including NHEK (Normal Human Epidermal Keratinocytes), HUASMC (Human Umbilical Artery Smooth Muscle cells), and so on, can undergo pyroptosis which is induced by chemotherapy drugs. Secondly, they demonstrated that after peritoneal injection of Cisplatin, wild-type mice had more severe destruction of the crypts and the villi than GSDME^-/-^ mice, and with the delivery of Cisplatin into lungs of WT mice caused alveolar wall thickening, neutrophil infiltration, and vascular injury. In addition, Cisplatin reduced the weight of WT mice by about 15%. After peritoneal injection of 5-Fu, WT mice had hemorrhage, inflammatory cell infiltration, and loss of the crypts in the small intestine. When bleomycin was administered to mice, WT mice developed more severe lung injury and inflammation than GSDME^−/−^ mice. GSDME-mediated pyroptosis can kill normal cells and increase the adverse effect of chemotherapy^5,36,74^. So, how to avoid the side effects of GSDME in the treatment of cancer is the focus of the next step.

## Conclusion

**Fig. 3:**
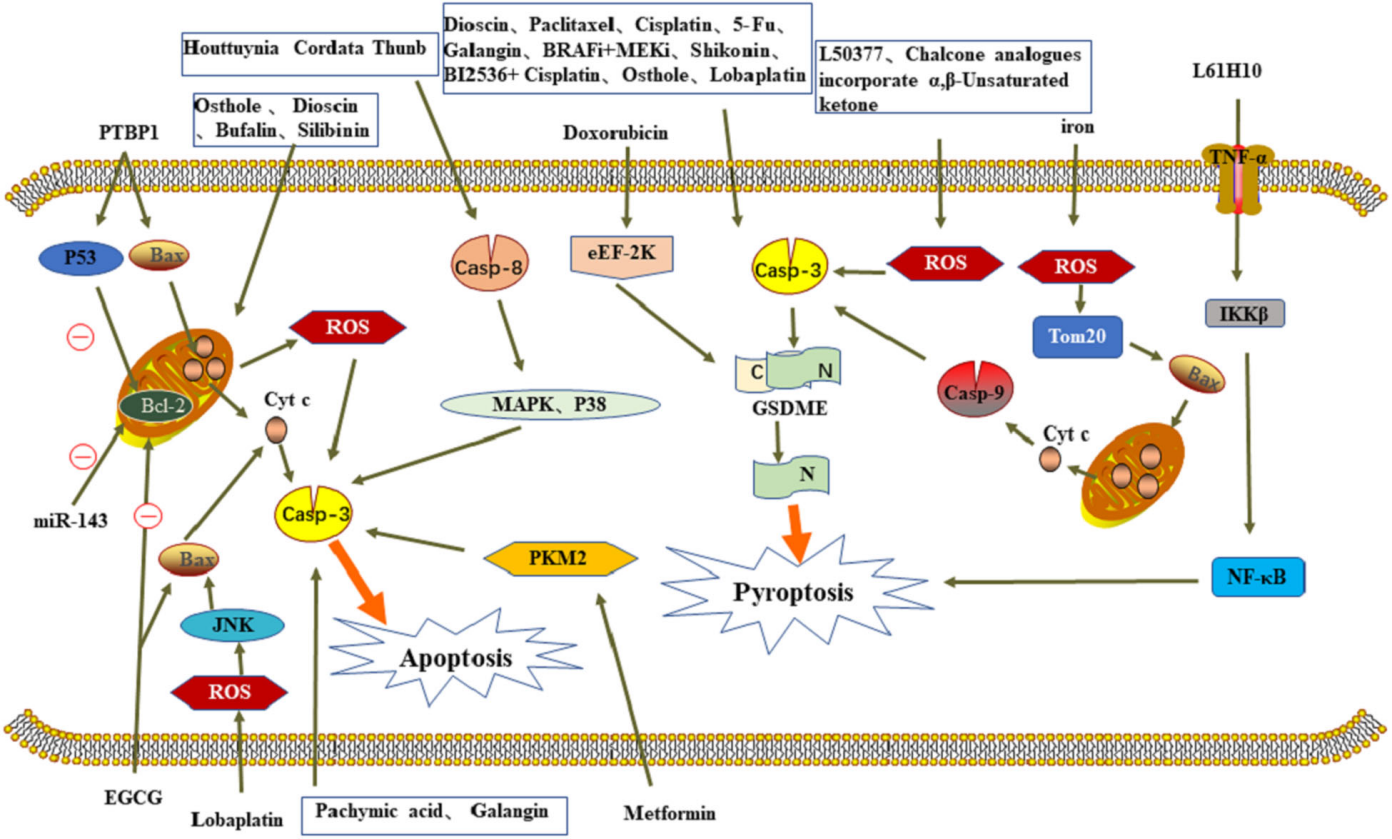
Graphical model of the effect of various molecules on caspase-3 dependent tumor cells death. The tumor supressor effect produced by these molecules uses caspase-3 as the hub to link apoptosis and pyroptosis.

Caspase-3-dependent apoptosis and pyroptosis can promote the removal of stressed, damaged, transformed or infected cells, and play a very important role in the development and treatment of tumors (Fig. 3). For a long time, apoptosis has been considered closely related to tumor treatment and prognosis. However, with the wide application of drugs, the anti-apoptotic ability of tumor cells is gradually enhanced. At the same time, pyroptosis, as a new inflammatory cell death mode, has entered the stage. Caspase-3 has been found to be a common key protein of apoptosis and pyroptosis in studies that aimed at improving cancer treatment with drug resistance. It is not only the terminal executor of apoptosis, but also has a pro-inflammatory effect. Therefore, caspase-3 can link apoptosis and pyroptosis, reducing the anti-apoptotic properties of tumors. The caspase-3/GSDME signal pathway as a “switch” that can shift the balance between apoptosis and pyroptosis in cancer. When GSDME is highly expressed, caspase-3 can cleave GSDME to trigger pyroptosis, otherwise, it triggers apoptosis. Although pyroptosis enhanced drug sensitivity, reduced the anti-apoptotic properties of tumors, and enhanced the anti-tumor immunity, high expression of GSDME could also increase the side effects of chemotherapy. And pyroptosis can release inflammatory cytokines that cause inflammatory carcinogenesis, but not enough to cause cancer progression. Therefore, there are still many problems for further study on caspase-3 and GSDME. Besides, studies have confirmed that caspase-3-dependent pyroptosis is secondary to necrosis after apoptosis. Furthermore, in tumor cells with low GSDME expression, the DNA methyltransferase inhibitor Decitabine, can inhibit the hypermethylation of its promoter to increase the expression of GSDME in tumor cells and induce pyroptosis. Above all, it has great research value for caspase-3/GSDME.

Our understanding of the role of caspase-3 in cancer is still limited, and the mechanisms of how caspase-3 affects cancer cells are not fully understood. The challenge for the future is to continue to understand the molecular details of caspase-3-dependent cell death and how these details improve the prospects for cancer treatment. We need to further elucidate the anti-cancer mechanism of caspase-3, deepen the understanding of the role of apoptosis and pyroptosis in tumor growth and proliferation, and facilitate the development of more effective anticancer drugs. How to find a balance point to utilize caspase-3/GSDME-mediated pyroptosis for cancer treatment is an open question and needs more further investigaions.
